# Young people’s views and experience of diet-related inequalities in England (UK): a qualitative study

**DOI:** 10.1093/heapro/daae107

**Published:** 2024-08-23

**Authors:** Vanessa Er, Mary Crowder, Eleanor Holding, Nicholas Woodrow, Naomi Griffin, Carolyn Summerbell, Matt Egan, Hannah Fairbrother

**Affiliations:** Department of Public Health, Environments and Society, London School of Hygiene and Tropical Medicine, 15-17 Tavistock Place, London, WC1H 9SH, UK; Sheffield Centre for Health and Related Research (SCHARR), University of Sheffield, Regent Court, 30 Regent Street, Sheffield, S1 4DA, UK; Sheffield Centre for Health and Related Research (SCHARR), University of Sheffield, Regent Court, 30 Regent Street, Sheffield, S1 4DA, UK; Sheffield Centre for Health and Related Research (SCHARR), University of Sheffield, Regent Court, 30 Regent Street, Sheffield, S1 4DA, UK; Population Health Sciences Institute, Fuse, Newcastle University, Ridley Building 1, Queen Victoria Road, Newcastle Upon Tyne, NE1 7RU, UK; Department of Sport and Exercise Sciences, Fuse, Durham University, Green Lane, Durham, DH1 3LA, UK; Department of Public Health, Environments and Society, London School of Hygiene and Tropical Medicine, 15-17 Tavistock Place, London, WC1H 9SH, UK; Faculty of Health, University of Sheffield, Barber House Annexe, 3 Clarkehouse Road, Sheffield, S10 2HQ, UK

**Keywords:** young people, health inequalities, diet, food environment, qualitative study

## Abstract

Inequalities in diets contribute to overall inequalities in health. Economic inequality and inequalities in access to healthy food are key drivers of poor diet and ill health among young people (YP). Despite mounting evidence of structural barriers to healthy eating, less is known about how YP view and experience these inequalities where they live, and how to address them. To explore YP’s perspectives on the drivers of diet-related health inequalities, we conducted three interlinked focus groups with YP aged 13–21 years from six youth groups across three geographical areas in England. We analysed the data inductively and deductively using reflexive thematic analysis and generated themes by examining how social structure, context and agency interact and impact YP’s diet. YP were aware of how inequalities in employment conditions impact their families’ income and ability to eat a healthy diet. They cited the high availability of hot food takeaways in their local areas as a significant barrier to healthy eating but did not support closing or restricting these outlets. They held strong views on policies to tackle diet inequality and showed a nuanced understanding of the strengths and limitations of universal and targeted approaches. Our study showed that YP have an awareness and understanding of food as important in relation to health, and of diet-related inequalities. However, further efforts are needed to shape and promote policies that resonate with YP and address both their health and wider social concerns.

**Contribution to Health Promotion**

- Our study recognizes that young people have an awareness and understanding of food as important in relation to health, and of diet-related inequalities.
- Young people have a nuanced appreciation of bounded agency: that is, the way social, cultural and economic factors shape individual food choices and practices.
- Young people are potential sources of support for health equity strategies that include social determinist approaches.
- Further efforts are needed to shape and promote policies that resonate with young people and reflect and address both their health and wider social concerns.

## BACKGROUND

Inequalities in diets contribute to overall inequalities in health (The Parliamentary Office of Science and Technology, 2022). Improvements in diets can improve population-wide health and reduce wider health inequalities. Poor diet in childhood and adolescence tracks into adulthood (Hovdenak *et al.*, 2019; Appannah *et al.*, 2021) and is associated with lower health-related quality of life (Wu *et al.*, 2019) and higher risk of chronic diseases, such as cardiovascular disease (Daniels *et al.*, 2011), diabetes (Lascar *et al.*, 2018) and some cancers (World Cancer Research Fund and American Institute for Cancer Research, 2018). According to the Global Burden of Disease dashboard, poor diet was the cause of 7.9 million deaths worldwide in 2019, accounting for 14% of all deaths (Global Burden of Disease Collaborative Network, 2020).

One of the main drivers for poor dietary quality is economic inequality and the relatively high cost of eating a healthy diet. According to the Food and Agriculture Organization’s (FAO) report on ‘The State of Food Security and Nutrition in the World’, more than 3.1 billion people across the world could not afford a healthy diet in 2021 (Food and Agriculture Organization *et al.*, 2023). In the UK, The Food Foundation reported an increase in the proportion of food insecure households with children, from 12.2% in 2022 to 24.4% in 2023 (The Food Foundation, 2023). Additionally, healthy nutritious food was two times more expensive than unhealthy products. Young people (YP) from disadvantaged backgrounds are more likely to experience food insecurity (O’Connell *et al.*, 2019), which has worsened since the COVID-19 pandemic and the cost-of-living crisis. Studies conducted in England have shown that YP from socioeconomically disadvantaged backgrounds are more likely to have poor diets (Johnson *et al.*, 2018; Public Health England and Food Standards Agency, 2021). The 2020–21 UK National Diet and Nutrition Survey revealed higher consumption of sugar-sweetened beverages and energy-dense food, and lower consumption of fruits and vegetables among the most socioeconomically deprived YP (Public Health England and Food Standards Agency, 2021). Furthermore, the latest statistics from the UK National Child Measurement Programme revealed that children aged 10–11 years living in the most deprived areas were more than twice as likely to be categorized as obese, based on body mass index, compared to those living in the least deprived areas (31.3% vs. 13.5%) (Office for National Statistics, 2022).

Another driver of YP’s diet is the neighbourhood food environment. There is consistent evidence showing a high density of fast-food and takeaway outlets in socioeconomically deprived areas and areas with a high concentration of ethnic minority population (Fleischhacker *et al.*, 2011; Molaodi *et al.*, 2012; Thornton *et al.*, 2016; Public Health England, 2018; Sanchez-Vaznaugh *et al.*, 2019). This means greater availability of, and access to unhealthy food, as these outlets tend to sell relatively cheap, energy-dense and nutrient-poor food (Jaworowska *et al.*, 2014; Huang *et al.*, 2022; Rinaldi *et al.*, 2022). The inequalities also extend to unhealthy food advertising where YP from ethnic minority and socioeconomically disadvantaged backgrounds are disproportionately targeted where they live, as well as online (Backholer *et al.*, 2021). In the UK, children living in low-income households are more likely to eat takeaway meals at home, and those who consume takeaways more frequently have poorer diets (Adams *et al.*, 2015; Taher *et al.*, 2019). The density of takeaway outlets across England increases each year (MRC Epidemiology Unit, University of Cambridge, 2019). A study of takeaway outlets in Norfolk, UK revealed that the density of takeaway outlets grew between 1990 and 2008, and the increase in the number of outlets was higher in the most deprived areas compared to the least deprived, with this widening over time (3.5 times higher in 2008 vs. 2.8 times higher in 1990) (Maguire *et al.*, 2015). There is emerging evidence of socioeconomic patterning in online access to takeaway outlets too. A recent study by Keeble *et al.* (2021) found that the percentage of registered food outlets on an online food delivery service in the most deprived areas was approximately two times greater than in the least deprived areas in the UK.

Despite mounting evidence of the structural barriers to eating a healthy diet, such as economic inequalities and the food environment, less is known about how YP view and experience these inequalities where they live, or how to address them. There have been two previous systematic reviews and a scoping review of YP’s views on healthy eating, all focusing on body size (e.g. obesity, body shape and weight) rather than health or health inequalities *per se*. One review included 11- to 16-year-olds (Shepherd *et al.*, 2006); another 12- to 18-year-olds (Rees *et al.*, 2014) and the third 18- to 24-year-olds (Munt *et al.*, 2017). None of these reviews presented detailed analysis focused on health inequalities, as the reviewers noted that included studies typically reported few details on equity dimensions. The reviews by Shepherd *et al.* (2006) and Rees *et al.* (2014) found that YP tended not to frame food as a health issue. Rather they tended to view food in terms of what they liked and disliked.

The case has been made that low agency population interventions, such as free school meals (FSMs), advertising restrictions of foods high in fat, salt and sugar (HFSS) and restrictions of takeaway outlets, are effective and equitable ways to reduce poor diet in the population (Adams *et al.*, 2016). However, research literature is sparse on YP’s views of such interventions, or their views on alternative (high agency) education interventions or the relative merits of targeted and population-level approaches. In England, the FSM (Department for Education, 2018) and holiday activities and food programmes (Department for Education, 2022) provide healthy food to school-aged children from low-income backgrounds, but there is no provision for older YP (e.g. aged 18–24) from similar backgrounds. Currently, there is a restriction on broadcast advertising for HFSS foods, but only for those aimed at children (UK Advertising Standards Authority, 2024). The existing planning policy only restricts the opening of new takeaway outlets (Keeble *et al.*, 2019), which has been perceived as less useful in areas that already have a high density of takeaway outlets.

To address the gap in the literature, our study was guided by the following questions:

1. What is YP’s understanding and experience of diet-related health inequalities?
2. What are the drivers of diet-related health inequalities where YP live?
3. What are YP’s views on addressing diet-related health inequalities where they live?

## METHODS

### Approach

The research in this article drew on data from a wider study exploring YP’s perceptions of what influences their opportunities to be healthy within their local area and their understanding of health inequalities (Fairbrother *et al.*, 2022). The philosophical underpinning of our approach is critical realism. We view health inequalities as real (they exist independently of human practices and awareness) while acknowledging the role of human practices, perspectives and social context in shaping how we know about health inequalities. In other words, our knowledge of health inequalities is subjective and incomplete. Our approach was critical in orientation (Braun and Clarke, 2024) in that, we sought to unpack and interrogate participants’ accounts to provide causal explanations of health inequalities and make recommendations that are relevant and beneficial to YP. We conducted a qualitative study to understand YP’s perceptions and experiences of health inequalities, with a focus on explaining the causal mechanisms of health inequalities.

### Positionality

As a team of public health researchers, our work is rooted in explaining and addressing health inequalities. Based on our knowledge of the literature, we assumed that YP have a more individualistic explanation of the causes of, and solutions to health inequalities. We believe that YP have a right to good health and they should be supported to live to their fullest potential. We made a conscious effort to include disadvantaged and marginalized voices. However, we recognized the power imbalance as we are older, well-educated and ‘relatively advantaged’ (though this term masks some variation in the research team, such as variation in social backgrounds, job security, housing security and other intersecting equity dimensions). Therefore, we took a participatory approach to research in our attempt to balance power dynamics and meaningfully include YP of disadvantaged backgrounds in discussions on health inequalities. Engaging in a process of reflexivity, we acknowledge that our analysis is informed by our prior understandings of health equity, including pre-held assumptions that social determinants, as described by Marmot *et al.* (2020), are particularly important for both explaining and tackling health inequalities. We acknowledge that our research findings extend from our subjective experience of the research, influenced by our social and professional backgrounds, and our particular interactions with the YP who participated.

### Sampling and recruitment

We recruited YP aged 13–21 years through six youth groups across three geographical areas in England; London, South Yorkshire and North East. Our original sampling frame targeted YP living in areas with contrasting levels of deprivation and geography (e.g. rural and urban). This was hampered by the COVID-19 pandemic, so we recruited YP from youth groups with whom we had established relationships, all of which were located in areas in the most deprived quintile based on the 2019 English indices of multiple deprivation. Furthermore, while we initially aimed to work with YP aged 13–17 years, we took an inclusive approach as some of our youth groups also included YP over 18.

### Data collection

We conducted a series of three interlinked focus groups with YP from each of the six youth groups between February 2021 and June 2021, resulting in a total of 18 focus group sessions. The focus groups were planned to be in-person, but we switched to an online format for all but three sessions with one youth group in the North East due to the UK’s lockdown and social distancing restrictions during the COVID-19 pandemic.

Session 1 used a participatory concept mapping activity (Jessiman *et al.*, 2021) to explore perceptions of what influences YP’s opportunities to be healthy in their local area. Session 2 examined YP’s understanding of health inequalities through prompted discussion of selected health-related news headlines, including FSMs, fast food and advertising of less healthy food. Session 3 focused on YP’s priorities for change to improve health in their area.

Facilitators used a topic guide (see Supplementary Material) that had been piloted with youth organizations to aid discussions. At least two researchers facilitated each focus group, accompanied by a youth worker for safeguarding purposes and to support YP if required (Woodrow *et al.*, 2022). Focus groups lasted between 90 and 100 minutes and were audio-recorded with consent. Participants also provided information on ethnicity, age and residential postcode (used to determine area deprivation level). We gave participants £20 vouchers at the end of each focus group as a token of appreciation for their time.

### Ethics

The study has ethics approval from the School of Health and Related Research (SCHARR) Ethics Committee at the University of Sheffield (ref: 037145). All participants provided written consent. For participants under the age of 16 years, opt-in consent was also obtained from parents/guardians.

### Data analysis

Prior to analysis, audio recordings were transcribed verbatim and anonymized by approved transcription services. The research in this article drew on data from a wider study exploring YP’s perceptions of what influences their opportunities to be healthy within their local area and their understanding of health inequalities (Fairbrother *et al.*, 2022). Here, we only include discussions about food as data. We analysed the data using reflective thematic analysis (Braun and Clarke, 2022) as it is theoretically flexible and fits with a critical realist approach. Using this approach, we examined the mechanisms and structures that give rise to diet-related inequalities by focusing on participants’ accounts and situating them within the contexts (realities) that participants live in. This requires continual reflexivity and critical engagement with the data and the analytical process.

V.E. and M.C. read the transcripts and applied a mix of semantic (surface meaning) and latent coding (underlying meaning), aided by a qualitative analysis software, NVivo 12. During the coding process, V.E. and M.C. met regularly to discuss the meaning of the data to ensure reflexivity and expand the interpretation of the data. We analysed the data deductively by using two frameworks as a lens to make sense of the data: Smith and Anderson’s (2018) framework for lay perspectives of socioeconomic health inequalities, and Pearce *et al.’s* (2019) conceptual model of pathways to inequalities in child health. We combined it with inductive analysis as we were open to the possibility that the data may not fit these frameworks.

Upon reflection, we decided that Giddens’s (1989) structuration theory which posits social practices as an interplay between agency and social structure, was a better fit for the data and used it to inform the conceptualization of themes. We focused on how diet-related inequalities were produced, by contextualizing YP’s eating practices and interactions with their local food environment, and connecting it with the social history and structure of the area where they live. The themes were further developed by V.E. and M.C. alongside discussions with C.S. and H.F. to ensure the themes capture the central meanings and patterns identified from the data and answer the research questions. V.E. wrote a narrative for each theme, with the scope of each theme being iteratively defined and refined with inputs from M.C., C.S., M.E. and H.F.

## RESULTS

Our final sample consisted of 42 YP aged 13–21 living in urban and rural areas, and of different genders and ethnicity (see Table 1).

**Table 1:** Participant characteristics

	London (*n* = 13)	South Yorkshire (*n* = 14)	North East (*n* = 15)
**Age range (years)**	16–21	15–17	13–20
**Gender (*n*)**			
Female	10	6	2
Male	3	7	9
Non-binary	–	–	2
Gender-fluid	–	1	–
Trans male	–	–	2
**Ethnicity (*n*)**			
White British	1	14	15
Asian/Asian British	6	–	–
Black/Black British	3	–	–
Mixed/Multiple ethnic group	2	–	–
Chinese	1	–	–

The YP in this study perceived eating a healthy diet as unattainable due to intersecting inequalities that manifested in their daily food practices and environment. YP’s agency to eat healthily was constrained by structural inequalities, mainly economic inequality and low availability and access to healthy food in deprived areas. We identified three key themes: (i) impact of economic inequality on family food practices, (ii) availability and access to hot food takeaways in areas of high deprivation and (iii) making healthy food more accessible to families.

(i) Perceived impacts of economic inequality on family food practices

This theme captures YP’s understanding of economic inequality. They viewed economic inequality through a ‘place’ lens, whereby some regions or neighbouring local areas in the UK were viewed as more economically disadvantaged than others. A recurring view suggested a mechanism whereby places with economic problems had poorer employment opportunities for YP and their parents. This in turn led to two types of barriers to healthy eating. Firstly, low incomes—YP believed that healthy foods tend to be more expensive than unhealthy food and that low-income families may have to choose between healthy diets and other essentials such as heating and school uniforms. Secondly, YP perceived that for many low-income families, there is less opportunity to prepare healthy meals at home due to a lack of time as a result of working long hours and/or multiple jobs.

Though YP acknowledged the importance of nutrition knowledge and cooking skills for healthy eating, they were acutely aware of how family income (or lack of it) restricted their ability to have an adequate and healthy diet.

*I see it sort of like if you have a run down job, you don’t have as much pay to pay for the food. Meanwhile, if you have a high job and you have the high society, you have more pay, therefore you’re able to take on more food. Which brings in the inequality with food discussed tonight.* (North East Group 2, Session 2)

There was a common perception among YP that unhealthy food is cheap while healthy food (described mainly as fresh fruits and vegetables) is expensive and thus unattainable on a low income.

*things like salads they’re expensive man, like five, six quid then the opposite of that, like portion of chips is like a quid…Even the prices of fruit and veg, I don’t know why they price up a bit too much.* (London Group 1, Session 1)

*I’d say so because like there’s a lot of income inequality where we are especially, so a lot of like poorer households find it hard to buy the more healthy stuff. They tend to be more expensive, especially in supermarkets…* (South Yorkshire Group 2, Session 1)

YP also knew of low-income families having to prioritize household bills and expenses over food. A few spoke from experience about their parents having to spend less on food to pay for essentials such as school uniforms. The need to make trade-offs came to the fore while discussing the impact of the COVID-19 pandemic. YP noted that some in their community could hardly afford to pay utility bills and were thus unable to store and/or cook fresh food, resorting to the convenience of fast-food or hot food takeaways.

*I think, with fast food, it doesn’t need to be maintained in a sense, so like, let’s say you can’t afford electricity bills, you can’t afford to keep your fridge running, or something. Buying healthy stuff isn’t, it’s not going to last, so just buying fast food, might be cheese and everything, is like OK, I’ve bought it, I can eat it, through it in a day, like it’s finished.* (London Group 1, Session 2)

*…that goes back to the budgeting thing. Some people, especially people who don’t necessarily have a lot of money might want to spend less on food and more on making sure that their child has the right stuff for school.* (South Yorkshire Group 1, Session 1)

While YP made direct reference to low income as a barrier to healthy eating, they also demonstrated an in-depth understanding of inequalities in employment conditions faced by those working in low-paid jobs, and how that negatively impacts one’s ability to eat a healthy diet. They pointed out that those in low-paid jobs often lack time to prepare and cook meals as they tend to work long hours or multiple jobs. The North-South (England) divide in economic opportunities was regularly brought up by YPs from the North East. According to one YP, movement restrictions during the COVID-19 pandemic further highlighted the inequalities experienced by those in low-paid jobs (e.g. bus drivers and delivery drivers) as they were less able to work from home.

*We did talk a bit about how people in the North, the sort of jobs that we have, it’s less likely that you’ll be able to work from home. So if you are working from home – which predominantly, especially if you’re in the South because a lot of the economies, they’re very knowledge-based – you can afford to do that sort of thing from home…So they have certainly got more time and more time that they can dedicate to something like cooking.* (North East Group 1, Session 2)

(ii) Availability and access to hot food takeaways in areas of high deprivation

This theme describes how YP felt physically surrounded by hot food takeaways in their local neighbourhood, and digitally surrounded via food delivery apps. They showed an understanding of how different elements of underserved communities intersect to encourage negative health practices.

YP cited the high density of hot food takeaways in their local area and a lack of food retail shops selling affordable and healthy options as barriers to healthy eating. As a YP related:

*I wanted to talk about like fast food joints, like in [Name of location] like there’s a lot of like fried chicken shops, a lot of like and they’ve renamed themselves to grills…like to get like more consumer support. And basically like, what I was saying was like it makes it harder for me.* (London Group 2, Session 1).

In terms of family food practices, the constant exposure to hot food takeaway was perceived as offering easy access to food for those whose home situations made it challenging to prepare home-cooked meals. For example, YP described how parents in low-income families worked long hours, leading YP and their families to take the ‘easier option’ of purchasing and consuming readily available hot food takeaways. Even though they were aware that hot food takeaways are unhealthy, they felt that they had to choose convenience over the nutritional quality of their meals.

*Because it could be quite a busy job that involves travel where they’d be an air hostess or a conductor for a train. It just might take a lot of time away from their family, so that has to force them to do – in a way – irrational things. Such as constantly sending a fast food order instead of healthy objects.* (North East Group 2, Session 2)

*Like if you come from a lower, like a poor area, maybe – like I knew someone who his mum would give him a quid and he’d go to the shop and just buy something and have it for his tea and he’d be out all night until like 10. His upbringings, obviously it’s not going to be the same as someone who has home-cooked meals every night that are prepared with nutritional value in mind.* (South Yorkshire Group 2, Session 3)

YP also talked about how the high availability of cheap and ‘tasty’ hot food takeaways, particularly around the school vicinity, made it convenient for YP to purchase and consume them. It was clear during the discussions that hot food takeaway outlets had become part of the social fabric of local life even though YP were critical of the ubiquitousness of these outlets where they live. They were considered by some YP as the ‘place to go’ after school as there was not much to do for YP in their local area, which also highlighted the lack of services and facilities that cater to youths in areas of high deprivation. Inadvertently, YPs socialized at hot food takeaway outlets and ended up consuming food deemed to be less healthy despite their intentions.

When probed about the high density of hot food takeaways, a YP highlighted the complex interdependency of individual and structural factors that affect one’s ability to eat healthily. Specifically, they explained that demand for cheap and quick meals resulted in the opening of hot food takeaway outlets, which in turn created high visibility and high consumption. This then reinforced the need for more outlets. In contrast, the cycle could be broken or disrupted by the lower demand for ‘cheap’ hot food takeaways in more affluent neighbourhoods.

*And then I guess because I think, like, yeah, if you’re in an area where demand is higher you’re going to have more takeaway so then if you’ve got more takeaway then that’s, kind of, what you see most of the time then you’re going to end up going to those takeaways more maybe. And seeing that, like, seeing it as more of an option compared to in a wealthier area where if at first you’re not, like, if there isn’t too much demand for fast food in wealthier areas then the takeaways aren’t really going to go there and then, because, like, and it is then, it is also easier to just go to the supermarket and, like, get stuff.* (London Group 2, Session 3)

YP’s narratives also suggested that hot food takeaways have a prominent online presence in their everyday lives. They related their experience of being inundated with advertising of HFSS sold by hot food takeaways in their local area when using food delivery applications, and though healthier options were available, they were more expensive and deemed unaffordable.

*…there’s about, I think it’s about 20 on my app just around me, because I live near a load of take aways.* (South Yorkshire Group 1, Session 2)

*…and like delivery companies, some of them actually offer the, the opportunity to like buy healthier options. Some of them do salads and all sort of meals that are meant to be healthier. But it’s like those options are very expensive, compared to the junk food, so called junk food options. So it still leaves you with no choice than to go for the junk food rather than the healthier option.* (London Group 1, Session 2)

(iii) Making healthy food more accessible to families

This theme presents YP’s views on public policies or interventions to address inequalities in accessing healthy food. YP had strong views on policies to tackle food inequality and showed a nuanced understanding of the strengths and limitations of universal and targeted approaches. Discussions about interventions, especially those targeted at low-income families were strongly tied to the stigma of poverty. YP in general were not in favour of a targeted FSM approach as they perceived it to be stigmatizing. As recounted by a participant: ‘*There’s an element of shame to it as to whether or not you will accept for yourself that you need that help, feeding your kids and feeding your family’ (North East Group 1, Session 2)*.

Although YP from low-income families who were eligible for FSM appreciated the assistance from the government, their accounts of receiving FSM referred to shame and centred on who was ‘poor enough’ to receive government assistance. One YP felt guilty for receiving FSM because their parents held ‘decent’ jobs (i.e. perceived to be well-paid) with full-time employment, and did not consider themselves to be impoverished.

*My mum’s a teacher and my dad works at the NHS, but a couple of years ago we were eligible for free school meals because it was something like me mum wasn’t earning enough so we therefore qualified. We felt kind of guilty, like we were robbing it from someone… because there are literally people on it who are choosing between feeding the kids at lunch or clothes for school uniform.* (South Yorkshire Group 1, Session 2)

One focus group referred to food voucher schemes, of the kind administered by some schools during the COVID-19 epidemic. Vouchers were seen as a way of giving families a way to decide how best to meet dietary needs and food preferences. This came up while discussing FSM provision during the COVID-19 pandemic, whereby most schools provided food parcels to students. There were strong criticisms of the quality of food parcels as the items were not nutritious and were overpriced. In contrast, another YP who received food vouchers shared how that gave her family the freedom to purchase food according to their needs, and thus they were able to maximize the value of the vouchers:

*So for instance, I get them (food vouchers) and we go shopping every month so we just save up all the vouchers and spend it on different places…We get tinned vegetables, like peas and carrots and that, but we don’t really like fresh veg or owt like that because it runs out of date really quickly. There’s no point really getting it.* (South Yorkshire Group 1, Session 2)

Most YP demonstrated empathy for those who were perceived to be worst off, for example, those who had to use food banks. Alongside feelings of shame and guilt for receiving FSM, YP shared concerns about the proliferation of food banks in their area and the stigma associated with going to one, particularly the fear of parents being blamed for their inability to provide food for their children. YP’s empathy also extended to local business owners. Although they recognized the adverse impact of hot food takeaways, YP did not want these outlets to be closed or restricted by local authorities—a planning policy that can be introduced by local authorities to limit the number of hot food takeaways, especially within the school vicinity. They expressed concern about the potential loss of income for the businesses and more importantly, loss of employment for workers at the outlets. In terms of supporting customer choice, YP often framed this as wishing to see healthier food options at local outlets as this is where they felt the choice was limited. Incentivizing customers to purchase healthier meals through a loyalty scheme was one of the examples given by YP.

*And, I don’t know, I was thinking incentives so, like, in a fast food chain, because you can’t shut them and you don’t really want to disturb their business, but if there was something, like, if you buy a certain number of the healthier meals and you get one of the less healthy meals for free or something, kind of, like a loyalty card…because I don’t think awareness alone necessarily helps because I think people are generally aware but it’s a case of actually, like, putting that into action and I think that can be quite tricky.* (London Group 2, Session 3)

## DISCUSSION

This article examines YP’s views on and experiences of inequalities in relation to their access to healthy food and diets. While previous studies have explored YP’s views of healthy eating (Shepherd *et al.*, 2006; Rees *et al.*, 2014; Munt *et al.*, 2017), there is little evidence on YP’s views of how inequalities in healthy eating occur and how to address them.

The YP we spoke to viewed inequalities in food and health partly in terms of how different people were more or less likely to consume healthy food (with fast food often used by participants as an archetypal ‘unhealthy’ food type). YP discussed inequalities in more explicit food poverty terms: showing awareness that some individuals and families struggle to afford sufficient food. Low income, coupled with the high cost of healthier food products, was singled out as a significant barrier to eating a healthy diet by YP. This is supported by analyses of food costs in the USA, UK and Europe (Kern *et al.*, 2017; Penne and Goedemé, 2021; The Food Foundation, 2023), which demonstrated that the cost of healthier food products was higher than less healthy products (two times more in the UK). Furthermore, one in five households in the UK would have to spend almost half of their disposable income to achieve a healthy diet, leaving little for other expenditures (The Food Foundation, 2023). YP also linked low income to inequality in employment opportunities, specifically low access to, and availability of higher paying jobs where they live, and its impact on their diet. This was raised by participants in all three study sites, but more prominently in the North East of England, which has one of the highest rates of unemployment and proportion of benefit claimants (Office for National Statistics, 2023b), and the lowest average weekly salary in England (Office for National Statistics, 2023a).

We found that YP in our study had a less individualistic understanding of inequality than was suggested in the literature (Backett-Milburn *et al.*, 2003; Vromen *et al.*, 2015; L’Hôte *et al.*, 2018; Smith and Anderson, 2018). While acknowledging the importance of dietary knowledge and cooking skills, our study participants showed an understanding of how structural inequalities impact their ability to acquire and consume a healthy diet. For example, YP were able to articulate the reinforcing connection between availability (supply) and purchase (demand) of hot food takeaways in their local area, which in turn made it easier for them to consume energy-dense and nutrient-poor food. The literature supports YP’s view that disparities in both physical and online food availability reinforce area inequalities. The high density of affordable but less healthy hot food takeaway outlets in deprived areas, both physically and online, has been well-documented (Fleischhacker *et al.*, 2011; Maguire *et al.*, 2015; Keeble *et al.*, 2021). There is also evidence that it is often the same communities who experience both low income and high exposure to fast-food takeaway in the UK. Burgoine *et al.* (2018), for example, demonstrating the double burden of low income and exposure to fast-food takeaway and its impact, found that the lowest income combined with the highest fast-food outlet proportion was associated with greater odds of obesity (odds ratio = 2.43, 95% confidence intervals: 2.09, 2.84). Another study in the UK found that within 4 years of Gateshead Council’s ban on planning permission for fast-food outlets, there was a 13.88% reduction in the proportion of fast-food outlets compared to five other local authorities in the North East of England which did not implement the ban (Brown *et al.*, 2022). The ban was associated with a decrease in the prevalence of overweight and obesity among children in year 6, living in areas that have a high density of fast-food outlets (Xiang *et al.*, 2022). This further demonstrates that obesity (as measured by body mass index) is a manifestation of social inequalities in health.

Previous reviews have found that health tends to be deprioritized in YP’s accounts of food, body shape and weight. The previous evidence suggests that YP focus instead on what they like and dislike, particularly on social factors such as social norms and peer expectations relating to body shape, and the social isolation that may result from not living up to those norms (Shepherd *et al.*, 2006; Rees *et al.*, 2014; Munt *et al.*, 2017). The previous reviews have little to say on health inequalities, perhaps reflecting YP’s apparent lack of interest in health *per se*. However, some reviewers pointed out that many authors of included studies neglected to provide data on health equity dimensions. In contrast, many of the YP we spoke to were willing to discuss food in relation to health and health inequalities. Possibly, this is because we (the researchers) made our interest in health inequalities known to participants, in contrast to some previously published studies where the researcher's interest in health inequalities may not have been apparent. However, we think it reasonable to hypothesize that health and inequalities may have been more present in YP’s minds at the time of our data collection, given that it occurred during the COVID-19 pandemic. The study also took place in an era where food poverty and food charity (such as food banks) have become more prominent in UK discourse (The Food Foundation, 2023).

Whilst we present YP’s interest in health inequality as a finding from our study, we are careful not to exaggerate the point. The YP we spoke to were also capable of discussing food availability and access in more social terms. For example, participants viewed fast-food chains as places where YP can socialize with friends. This is consistent with findings from a qualitative study of local (adults) perceptions and experiences of chicken shops in a deprived area in East London, which found the shops described as a part of everyday life and valued community spaces (Thompson *et al.*, 2018). Some participants of our study even felt protective towards local businesses, not wishing to see them shut down. YP’s attitudes towards measures intended to address food poverty were also shaped by more than food considerations. The thought of using welfare or charity was associated by some YP with stigma—and also guilt that seeking help to obtain food could prevent assistance from going to someone with a greater need. These attitudes present insights into what YP regard as social responsibility: supporting local businesses (even unhealthy ones), and assuming that charity and welfare are finite resources best targeted at those in greatest need.

YP’s attitude to universalism was complex. Some appeared to support universal approaches, for example, universal FSMs as a means of reducing stigma. Some wanted further intervention from the government to extend the FSM funding to include after-school and holiday meals. This is in line with the findings of two studies with YP in the UK (Fairbrother *et al.*, 2012; Knight *et al.*, 2018) which emphasized government and corporate responsibility for ensuring adequate nutrition and a healthy diet is affordable for families. However, other studies on the public’s attitudes towards inequalities have revealed a reticence towards government intervention and a preference for educational (information) and individual behavioural change interventions instead (Backett-Milburn *et al.*, 2003; Vromen *et al.*, 2015; Smith and Anderson, 2018). A comparison study of YP’s view on inequality in the USA, UK and Australia, also found most participants focused on individualized (agency) explanations of, and solutions for inequalities, with little critical engagement of the structural causes of inequalities (Vromen *et al.*, 2015).

### Implications

Our findings show YP have a nuanced appreciation of bounded agency: that is, the way social, cultural and economic factors shape individual choice and practices. However, public health policymakers might view some of the YP’s views with mixed feelings. On the one hand, there is evidence of YP’s understanding of health inequalities, and social determinants, and a clear desire for improvement. On the other hand, this section of the public holds views on social responsibility that do not all fit neatly within the kind of universalist and regulatory approaches to health equity that many UK public health practitioners have long espoused (Bambra *et al.*, 2011; L’Hôte *et al.*, 2018; Marmot *et al.*, 2020). We found evidence of common ground between YP and public health viewpoints, but further bridging work between the public health community and the public is still required.

We believe that our study can contribute to a re-imagining and updating of the evidence base about YP’s views about food and inequalities. In contrast with previous evidence, this re-imagining recognizes that YP have an awareness and understanding of food as important in relation to health, and of diet-related inequalities—including considerations of both individual behaviour and social determinants. While different findings between studies may reflect methodological differences, there are also plausible reasons for hypothesizing that times have changed, and that YP’s views have changed with them. This hypothesis should be explored further in future research.

### Strengths and limitations

We recruited YP living in areas of high deprivation. Using a place lens coupled with participatory concept mapping was an accessible way of eliciting YP’s views on diet-related health inequalities. It made tangible the structural inequalities that manifest in everyday life and impact YP’s ability to eat a healthy diet. It also allowed us to explore YP’s diet in multiple aspects of their life and a range of settings, including school, home and community.

Though our study showed that YP’s agency of purchasing and consuming a healthy diet was constrained by individual and area-level economic inequalities, we were unable to explore how that differs by levels of autonomy and agency. A Lancet series on dietary intake among adolescents (aged 10–24 years) emphasized the need to view adolescents as unique; each with different development trajectories within diverse sociocultural contexts (Neufeld *et al.*, 2022), rather than being defined by age only. Most of our participants still lived at home and thus conversations about food centred around school meals and hot food takeaways. However, a few participants who had more independence and agency felt that they were being forgotten and not supported by existing policies to obtain and consume a healthy diet. In two of the three study sites, all participants were white British. Although these areas have a high proportion of White population, we may have obtained more diverse views if we had included YP of different ethnicity. There were mentions of value for money as a key factor influencing food purchases in our study. A deeper exploration would enhance our understanding of what value for money means to YP, and its implications.

## CONCLUSION

Individual and area-level economic inequalities constrain YP’s ability to eat a healthy diet. The YP we spoke to appeared to be aware of this. We hypothesize that this awareness may reflect changing contextual factors such as the experience of the COVID-19 pandemic and widely discussed concerns about food poverty. YP are potential sources of support for health equity strategies that include social determinist approaches. However, it would be a mistake to assume this support can be relied on without further efforts to shape and promote policies that resonate with YP and address both their health and wider social concerns.

## Supplementary Material

daae107_suppl_Supplementary_Material
